# Idiopathic pulmonary artery dissection: a case report

**DOI:** 10.4076/1752-1947-3-7426

**Published:** 2009-07-23

**Authors:** Khalid Mohammad, Mohammad Sahlol, Osbert Egiebor, Ruxana T Sadikot

**Affiliations:** 1 Department of Veterans Affairs, Jesse Brown VA Hospital, Chicago, IL, USA; 2 Cook County Hospital, Chicago, IL, USA; 3 Section of Pulmonary, Critical Care and Sleep Medicine, University of Illinois, Chicago, IL, USA

## Abstract

**Introduction:**

The occurrence of pulmonary artery dissection is extremely rare in patients without pulmonary hypertension, congenital cardiac abnormalities or cardiac intervention. A diagnosis of pulmonary artery dissection is rarely made during life because it generally leads to cardiogenic shock and sudden death. The progression or natural course of pulmonary artery dissection is not known and the optimum management is not defined because of the paucity of cases in the literature.

**Case presentation:**

We report a rare case of a 51-year-old female patient, without pulmonary hypertension or other cardiac abnormalities, who presented with acute chest pain and was found to have a pulmonary artery dissection.. The diagnosis of pulmonary artery dissection was confirmed by computed tomography scan of the chest and cardiac magnetic resonance imaging. The patient declined surgical intervention and was followed up closely with medical therapy. At almost a year after her initial presentation, the patient is stable with no complications.

**Conclusions:**

To our knowledge, there are no similar cases reported in the literature of people with pulmonary artery dissection who have been followed up and who have not had surgical intervention. We review the etiology, pathophysiology, clinical associations, diagnosis and management of patients with pulmonary artery dissection.

## Introduction

Dissection of the systemic arteries is a well recognized and often non-fatal consequence of essential hypertension; however, pulmonary artery (PA) dissection is extremely rare and is usually a lethal complication of chronic pulmonary hypertension [1]-[4]. Patients who present with dissection of the PA often have underlying congenital heart disease, idiopathic PA, hypertension or have received a cardiac intervention. PA dissection usually manifests as cardiogenic shock or sudden death and is therefore typically diagnosed at postmortem rather than during life [5]-[9]. There are sporadic reports of patients with PA dissection in the literature who present with acute chest pain during life. This patient is unusual as she remains stable almost a year after the diagnosis of the dissection without surgical intervention.

## Case presentation

A 51-year-old obese female patient with chronic obstructive pulmonary disease (COPD) presented to our facility with acute onset of retrosternal chest pain. She described similar episodes on and off for a year but she did not seek medical attention. Most episodes lasted for a brief period and resolved without any intervention. This episode was prolonged and hence she came to the emergency department. The patient denied shortness of breath, orthopnea or paroxysmal nocturnal dyspnea (PND) and her exercise tolerance was not limited because of COPD. On examination, she appeared in no distress with a respiratory rate of 18, blood pressure of 149/80, pulse rate of 80/minute with a normal oxygen saturation. Systemic examination was unremarkable.

**Figure 1:**
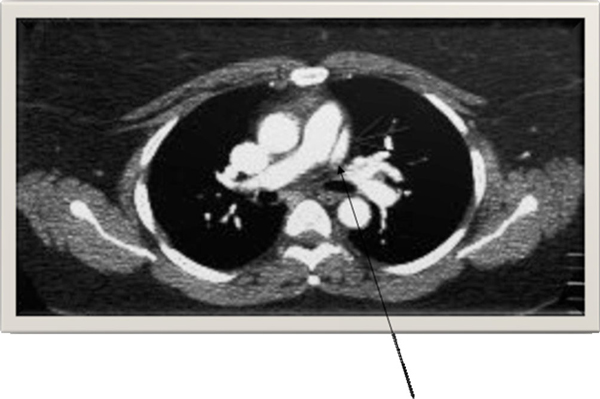
**Computed tomography of the chest with contrast showing a linear hypodensity within the main pulmonary artery suggestive of an intimal dissection flap of the trunk of the main pulmonary artery without dilation**.

**Figure 2:**
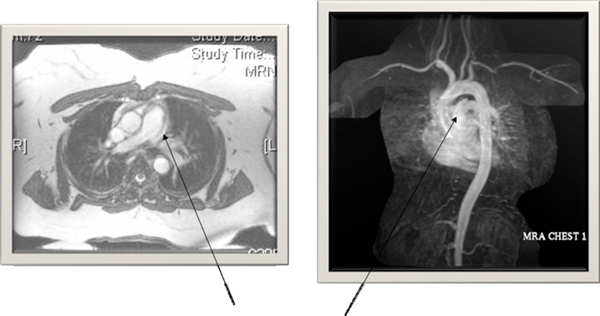
**Cardiac magnetic resonance imaging demonstrating an intimal dissection flap of the main pulmonary artery**.

Her initial investigations, which included complete blood count, renal function tests, electrocardiogram (EKG) and chest X-ray, did not reveal significant abnormalities. Cardiac enzymes excluded an acute coronary syndrome. Computed tomography (CT) of the chest with contrast was performed to rule out a pulmonary embolism and showed a linear hypodensity within the main PA (MPA) suggestive of an intimal dissection flap of the trunk of the MPA without dilation (Figure 1). A transthoracic echocardiogram showed a normal left ventricular (LV) systolic function, with normal flow through the right heart and pulmonary valve. PA pressures were normal and no valvular or congenital defects were identified. Cardiac magnetic resonance imaging (MRI) confirmed an intimal dissection flap of the MPA with no evidence of congenital heart disease (Figure 2). She was treated with oxygen and diltiazem and urgently referred to the cardiothoracic surgeons who offered her a surgical repair. The patient declined the surgical option because of its high risk nature. She was continued on diltiazem with a close follow-up. Interestingly, almost a year since her initial presentation, she remains stable with no further complications.

## Discussion

Dissection of the MPA is a rare event and is an unusual complication of chronic pulmonary hypertension which in most cases is associated with congenital cardiac abnormalities. The majority of patients present with cardiac shock or sudden death and hence the diagnosis is rarely made in living patients. Over the past two centuries, only 63 cases of people with PA dissection have been reported in the literature [8]-[10] of whom eight were diagnosed during life [9]-[17]. We report a case of idiopathic dissection of the MPA in a patient without pulmonary hypertension, cardiac disease or cardiac intervention who presented with acute chest pain.

Among the 63 previously reported cases of PA dissection, 34 patients had underlying cardiac disease most commonly congenital heart defects including patent ductus arteriosus and seven patients had rheumatic mitral stenoses. Nine patients with idiopathic PA hypertension with dissection have been reported. Two of these patients developed a dissection post lung [11] or heart lung transplantation [12]. In four patients, dissection was associated with pulmonary thrombosis. There are three cases that have been related to a cardiac intervention [15]-[17]. Although Marfan's syndrome is often associated with aortic dissection, so far there is only one reported case of PA dissection in a patient with Marfan's syndrome [1]. Interestingly, two patients were reported to have aortic and PA dissection which was not associated with Marfan's syndrome [18,19]. There are three previous cases that have been reported as idiopathic dissection and were hypothesized to be secondary to some inflammatory or non-specified cause [13]. The overall sex distribution among all of the reported patients appears to be equal with a wide age range from 26 days to 85 years. Peak incidence of dissection was in the third and sixth decades. In younger patients, congenital heart abnormalities were the most common underlying causes, whereas in older patients, diseases were associated with PA dissection.

The majority of PA dissections occur in the presence of medial degeneration with fragmentation of elastic fibers and generalized dilatation of the pulmonary arterial tree caused by chronic pulmonary hypertension [1]-[3,13]. With medial degeneration, the wall is weakened, the vessel may dilate, and the raised intravascular pressure and shear stresses may predispose to the development of an intimal tear. Whether medial degeneration causes the dissection, predisposes to intimal tears, or results from chronically raised intravascular pressure remains controversial. The main pulmonary trunk is the site of dissection in about 80% of patients, usually without involvement of the branches. Occasionally, an isolated dissection of the right or left PAs and intrapulmonary branches can occur [1]-[3,13]. In a small proportion of patients, PA dissection may occur at the site of localized aneurysm formation.

Over the past two decades, PA dissection has been diagnosed during life in seven of the reported patients [9]-[15]. This may reflect the technological advances and increased use of sophisticated modalities to make diagnosis of pulmonary vascular diseases. Non-invasive imaging methods such as echocardiography, CT, and MRI were used to detect PA dissection in most of these patients. Our patient's CT scan showed a linear hypodensity within the MPA which was confirmed on a cardiac MRI. The MRI and echocardiogram excluded other cardiac abnormalities and showed normal PA pressures. Among the living patients who have been diagnosed with a PA dissection, chest pain and dyspnea were the most common presenting symptoms.

The optimum management of patients with PA dissection has not been defined because of the low number of cases in the literature. Based on anecdotal reports, surgical repair has been performed in an occasional patient. Out of the reported patients that have presented emergently and that were diagnosed in a timely manner, three patients have had a successful intervention [13,14]. There is one previous report of a patient who was managed conservatively with diuretics and vasodilators although it is not known for how long the patient was followed up [20]. We offered an emergent thoracic surgery consultation to our patient for repair of the dissection. However, she declined surgical intervention and hence was closely followed up with medical therapy. Interestingly, she continues to do well with no further symptoms almost a year after her initial presentation. The reason for her stabilization can only be speculated. We believe that her dissection was partial, with intermediate healing, and did not involve the entire thickness of the PA.

## Conclusion

In cases where the dissection is related to pulmonary hypertension, there may be thinning of the arterial walls because of the high pressures in the arteries. Thus, the dissection may involve the entire thickness of the wall which leads to a catastrophic presentation with acute cardiogenic shock or death. Diagnosis of PA dissection is rarely made during life. Thus, the progression or natural course of PA dissection is not known. This case is extremely unusual as the patient remains stable without surgical intervention.

## Abbreviations

COPD: chronic obstructive pulmonary disease; CT: computed tomography; EKG: electrocardiogram; MPA: main pulmonary artery; MRI: magnetic resonance imaging; PA: pulmonary artery; PND: paroxysmal nocturnal dyspnea.

## Consent

Written informed consent was obtained from the patient for publication of this case report and any accompanying images. A copy of the written consent is available for review by the Editor-in-Chief of this journal.

## Competing interests

The authors declare that they have no competing interests.

## Authors' contributions

All the authors have contributed to the patient data collection. Drs Mohammad and Sadikot have contributed to the writing.
